# Editorial: Injuries, injury prevention and training in climbing - volume II

**DOI:** 10.3389/fspor.2025.1649613

**Published:** 2025-08-11

**Authors:** Gudmund Grønhaug, Atle Hole Saeterbakken, Volker Rainer Schöffl, Andreas Schweizer, Yasser El Sheik

**Affiliations:** ^1^ Hogskulen pa Vestlandet, Bergen, Norway; ^2^ Department of Orthopedic and Trauma Surgery, Klinikum Bamberg, Bamberg, Germany; ^3^ Universitatsklinik Balgrist, Zürich, Switzerland; ^4^ North York General Hospital, Toronto, ON, Canada

**Keywords:** climbing, sport medicine, injury prevention, bouldering, injury

**Editorial on the Research Topic**
Injuries, injury prevention and training in climbing - volume II

Climbing as a sport has matured. We are now seeing more evidence on systematic training and how to achieve an elevated level of performance, and research is closing scientific gaps regarding injury prevention and treatment. Appearance of the sport in two Olympics has changed the perspective of what is achievable in climbing. Still, for the vast majority, climbing is an unorganized recreational activity, making it impossible to quantify the total number of people attending climbing on a regular basis.

The increase in popularity of climbing is demonstrated in the introduction of research papers: authors no longer need to explain what climbing is, how it is organized, or how it has grown in recent decades.

The different disciplines of climbing demand different morphologies and training regimes and encompass different risk factors, resulting in different potential injuries. As climbing as a sport becomes more specialized, the research must follow along. It is now time for research on climbing to clearly distinguish between what discipline the respondents or subjects are training for. Furthermore, research results need to be presented with analyses based on gender and levels of performance. This way of presenting results and discussion will make the research easier to apply to relevant groups. Randomized controlled trials have rarely been included in climbing literature It is, therefore, great to see Saeterbakken et al. presenting a randomized controlled trial examining climbing training in this research topic. We hope to see more of these in the future.

The quality of the research on climbing has been improving over the last decade, with new areas, new perspectives, and new methodologies. The research is also becoming increasingly globalized. In this research topic, we are presenting eight papers from a total of 32 researchers representing most of the continents. This research topic covers a thematic span from how to optimize training, what to focus on while training, how to treat injuries, and potential barriers to seeking treatment as well as an insight on optimal body composition for bouldering.

A topic that has always been of importance in climbing research is what it takes to become a top-level climber. A never-ending debate in the world of climbing is the importance of height. Is it favorable to be tall or not? These questions and more are answered in a study of the morphology of the World Cup elite boulderers that compares them with national-level climbers and a group of students (Draga et al.). Some might find the results surprising while others will have their view confirmed.

Another topic that makes headlines is the relatively high level of eating disorders and potential Low Energy Availability (LEA) or Relative Energy Deficit (RED-S) among climbers. An interesting study is examining the possible interactions between social media use and food tracking behaviors (Slagel et al.). The authors recommended campaigns on social media to help climbers maintain a healthy body image to avoid potential health issues and injuries. Importantly, the study included recreational climbers, meaning the findings are not only related to high-performance athletes but everyday men/women you meet in the climbing gym or at the crag.

By presenting new research in a research topic, we can see several papers together. In this topic we can see the broader picture when combining the new research on what is the most important feature for predicting performance (Draga), how to perform training (Saeterbakken et al.), and how to optimize recovery (Krupková et al.). All these three papers are important in their own respect but give a deeper insight when read together as a total.

Injuries in climbing are sadly rather common and well documented in previous research. However, the novelty of the growing body of evidence is how to treat the injuries. In this topic we can present treatment algorithms for two common finger injuries in climbing. Examined a treatment algorithm for capsulitis (Schöffl et al.) and synovial chondromatosis (Becker et al.).

Sadly, climbers do not seek or use professional health care as often as they should. The lack of trust in health care seems to be a phenomenon that repeats itself across the globe. In this research topic, one of the studies examines the barriers for health seeking behavior in Manilla (Cruz and Cabrera). This paper is also one of the few that focuses on indoor climbing.

To sum up, we are proud to present the second edition of the research topic on Injuries, injury prevention, and training in climbing.

This research topic aimed to gain new insight into topics relevant for injury, injury prevention, and training methodology in the different disciplines of climbing. On behalf of the editorial team and with help from the researchers who chose to publish their research in this topic, I am proud to say that we achieved our goal.

We are aiming to open up a third volume of this topic in early 2026. In the next volume we hope to see new research to gain more insight into climbing injuries, injury prevention, and training. The research has the potential to help the sport of climbing to reach new heights and fulfil its potential, without injuries!

